# Characterizing the Piezosphere: The Effects of Decompression on Microbial Growth Dynamics

**DOI:** 10.3389/fmicb.2022.867340

**Published:** 2022-05-17

**Authors:** Anaïs Cario, Gina C. Oliver, Karyn L. Rogers

**Affiliations:** ^1^Department of Earth and Environmental Sciences, Rensselaer Polytechnic Institute, Troy, NY, United States; ^2^Rensselaer Astrobiology Research and Education Center, Rensselaer Polytechnic Institute, Troy, NY, United States

**Keywords:** *Desulfovibrio salexigens*, *Archaeoglobus fulgidus*, high-pressure microbiology, microbial physiology, deep marine biosphere

## Abstract

The extent to which the full diversity of the subsurface microbiome can be captured *via* cultivation is likely hindered by the inevitable loss of cellular viability from decompression during sampling, enrichment, and isolation. Furthermore, the pressure tolerance of previously isolated strains that span surface and subsurface ecosystems can shed light into microbial activity and pressure adaptation in these transition zones. However, assessments of the effects of elevated pressure on the physiology of piezotolerant and piezosensitive species may be biased by high-pressure enrichment techniques. Here, we compared two high-pressure cultivation techniques—one that requires decompression of the whole cultures during sampling and one that employs the previously described isobaric PUSH devices—to explore the effects of repeated decompression during incubations performed to characterize isolates from deep environments. Two model sulfate-reducing prokaryotes were used to test the effects of decompression/repressurization cycles on growth rates, cell yields, and pressure tolerance. The mesophilic bacterium *Desulfovibrio salexigens* was cultivated from 0.1 to 50 MPa, and the hyperthermophilic archaeon *Archaeoglobus fulgidus* was tested from 0.1 to 98 MPa. For both cultivation methods, *D. salexigens* showed exponential growth up to 20 MPa, but faster growth rates were observed for isobaric cultivation. Furthermore, at 30 MPa minor growth was observed in *D. salexigens* cultures only for isobaric conditions. Isobaric conditions also extended exponential growth of *A. fulgidus* to 60 MPa, compared to 50 MPa when cultures were decompressed during subsampling. For both strains, growth rates and cell yields decreased with increasing pressures, and the most pronounced effects of decompression were observed at the higher end of the pressure ranges. These results highlight that repeated decompression can have a significant negative impact on cell viability, suggesting that decompression tolerance may depend on habitat depth. Furthermore, sampling, enrichment, and cultivation in isobaric devices is critical not only to explore the portion of the deep biosphere that is sensitive to decompression, but also to better characterize the pressure limits and growth characteristics of piezotolerant and piezosensitive species that span surface and subsurface ecosystems.

## Introduction

Most of the bacterial and archaeal biomass on Earth is in deep-sea and subsurface environments at elevated pressures (Whitman et al., 1998; Kallmeyer et al., 2012; Parkes et al., 2014; Bar-On et al., 2018). These microorganisms have been shown to be well-adapted to the elevated pressures of their natural habitats (e.g., Somero, 1992; Allen and Bartlett, 2002; Simonato et al., 2006; Jebbar et al., 2015; Peoples and Bartlett 2017). Despite this, pressure seems to be one of the least explored parameters for microbial growth, as relatively few microorganisms from these deep environments have been isolated and/or grown under *in situ* pressures. To date, fewer than 100 species have been reported to be piezotolerant or piezophilic (Picard and Daniel, 2013; Jebbar et al., 2015; Cario et al., 2019; Oliver et al., 2020; Alain et al., 2021; Courtine et al., 2021; Li et al., 2021; Yu et al., 2021; Zhang et al., 2022), and few obligate piezophiles have been identified (Bartlett, 2002; Zeng et al., 2009). Therefore, our knowledge of active microbial species, their physiological and metabolic potential, and community diversity in these deep environments is limited.

Exploration of high-pressure life is limited by the difficulty in both sampling from high-pressure environments and replicating those pressure conditions in the laboratory during cultivation, isolation, and characterization. For obligate piezophiles, high-pressure sampling and cultivation is the only route to isolate novel species (Yayanos and Dietz, 1983; Jannasch and Wirsen, 1984). However, facultative piezophiles and piezotolerant microorganisms can often withstand lower sampling and transfer pressures (Jannasch and Wirsen, 1984). In these cases, the total change in pressure, duration of decompression, and number of subsequent decompression/repressurization cycles can impact the successful isolation of piezophiles (Yayanos, 2001; Park and Clark, 2002; Peoples and Bartlett, 2017). The pressure condition of cultivation experiments may also impose a selection bias on enrichment and isolation experiments that favor species more tolerant to decompression or lower growth pressures (Jannasch et al., 1992; Yayanos, 1995; Grossart and Gust, 2009). Furthermore, Park and Clark (2002) showed that rates of decompression could also impact microbial survival during decompression. For example, accelerated rates of decompression (26 MPa/s) caused the piezophile, *Methanococcus jannaschii* cells to rupture while slower rates of decompression (5.2 MPa/min) over the same pressure range increased viable cell yields (Park and Clark, 2002). Additionally, many piezophiles recovered from the intestinal systems of deep-sea macro fauna (Yayanos et al., 1979; Deming et al., 1981; Jannasch and Wirsen, 1984), show a greater tolerance to sample decompression, but the loss of species from decompression has yet to be quantified and any correlation with habitat depth has yet to be explored (Yayanos, 1978).

Deep biosphere species are often exposed to decompression during sample recovery, as well as during transfer, enrichment, and isolation. Therefore, significant effort has gone into developing pressure-retaining vessels to sample from the deep ocean habitats and carry out enrichment and isolation experiments without decompression (Tabor and Colwell, 1976; Jannasch and Wirsen, 1977; Yayanos, 1977; Cahet et al., 1990; Bianchi et al., 1999; Tamburini et al., 2003; Kato et al., 2008; McNichol et al., 2016; Cario et al., 2019). Such variable volume, floating piston devices can maintain elevated pressure during subsampling, inoculation, and/or transfer (Bianchi et al., 1999; Tamburini et al., 2003; Garel et al., 2019). Recent application of these new technologies has confirmed that higher rates of microbial activity and cell growth are achieved in incubation studies of bathypelagic samples maintained at *in situ* pressures without decompression, compared to parallel, decompressed, ambient pressure experiments (Tamburini, 2006; Tamburini et al., 2013; Garel et al., 2019, 2021). These high-pressure incubation experiments emphasize the need to study deep-sea microbes under *in situ* pressure conditions in order to accurately quantify deep-sea prokaryotic activity.

For enrichments and isolation, common methods involve growth in syringes, plastic bulbs, or heat-sealed plastic bags, in static pressure vessels (reviewed in Yayanos, 2001). However, subsampling to monitor cell growth over time requires decompression of the entire system followed by repressurization for continued cultivation. Inoculating such devices for high-pressure enrichment experiments also usually requires ambient pressure conditions. Other alternatives to study microorganisms under *in situ* pressure conditions include (i) the implementation of optic windows in the high-pressure vessels to monitor growth and motility (Maldonado et al., 2016; Garel et al., 2019); (ii) using high-pressure capillaries or other high-pressure cells coupled with microscopy to study molecular behaviors under extreme conditions with specific high-pressure cell using fluorescence microscopy (Raber et al., 2006; Usui et al., 2012; Patra et al., 2017; Bourges et al., 2020); and (iii) application of spectroscopy techniques to characterize microbial metabolism under high-pressure conditions (Kato and Fujisawa, 1998; Picard et al., 2007, 2015; Peters et al., 2014; Martinez et al., 2016; Osman et al., 2021). These alternatives are promising but require both specific and costly equipment and expertise to perform high-pressure *in situ* monitoring, and are not-yet widespread.

Here, we compare microbial growth in traditional static pressure vessels with cultivation in variable volume, floating piston isobaric high-pressure vessels to explore how decompression affects microbial growth patterns and the range of growth pressures at HHP for two model subsurface sulfate-reducing prokaryotes. Building upon previous designs (Bianchi et al., 1999; Tamburini et al., 2003), we recently collaborated with TOP Industrie© (Vaux Le Peńil, France) to develop a high-pressure, high temperature (HT; 100 MPa, 121°C), floating piston device with a 50 ml PEEK-lined reservoir (Cario et al., 2019). These pressurized underwater sample handler (PUSH) vessels were designed to sample from deep-sea environments and enable subsequent high-pressure enrichment and isolation without decompression (Oliver et al., 2021). These PUSH vessels were used to grow two sulfate-reducing prokaryotes, in high hydrostatic pressure (HHP) batch cultivation experiments that compared growth rates between cultures that experienced several cycles of decompression/repressurization, and those that were maintained at constant pressures throughout the experiment. High-pressure growth of *Desulfovibrio salexigens*, a mesophilic bacterium previously reported to be piezosensitive (Bale et al., 1997) was tested up to 50 MPa at 30°C (optimum temperature at 0.1 MPa). We have previously reported piezotolerant growth of *Archaeoglobus fulgidus* (Oliver et al., 2020), a hyperthermophilic archaeon, under high-pressure conditions with cycles of decompression/repressurization, and here explore growth at constant incubation pressures from 0.1 to 98 MPa at 83°C (optimum temperature at 0.1 MPa).

## Materials and Methods

### High Temperature, High Hydrostatic Pressure Equipment

#### Pressurized Underwater Sample Handler Vessel Batch Culture System

The PUSH vessels, similar to those vessels described in Bianchi et al. (1999) and Garel et al. (2019), and purchased through TOP Industrie©, were used for HT and HHP microbial batch cultivation without whole sample decompression and repressurization cycles during subsampling periods (Oliver et al., 2020). The PUSH vessels have a maximum pressure and temperature range of 0.1–100 MPa and 25°C–160°C, respectively, (as detailed in Oliver et al., 2021). For HT conditions, the temperature was controlled by a heating jacket and thermocouple system constructed for each PUSH vessel. The heating jackets, thermocouples, and temperature controllers were purchased from OMEGA™. Each vessel had an insulation wrap over the heating jacket and thermocouple. This heating system can be moved into an anaerobic chamber and plugged into a DC to AC converter powered by a 12-volt battery to maintain HT conditions during anaerobic preparations so that stable pressures could be reached upon pressurization (Supplementary Figures S1B,C). A decompression line was constructed to mitigate rapid pressure changes during subsampling that might induce cell shearing or death (Park and Clark, 2002; Foustoukos and Pérez-Rodríguez, 2015). The line included a micrometering valve to slowly subsample while operating the HHP screw pump to maintain vessel pressures (Supplementary Figure S1E).

#### Syringes in Static Pressure Vessels for HT-HHP Batch Cultures

Similar to several previous high-pressure growth experiments (e.g., Marteinsson et al., 1997; Takai et al., 2008; Oliver et al., 2020) syringes were used as reaction vessels in heated static pressure vessels, which were decompressed and repressurized at each sampling point. *Desulfovibrio salexigens* was grown in plastic syringes, and *A. fulgidus* was grown in glass syringes (Hamilton© 10 ml, 1,000 series luer lock gastight glass syringes) to maintain anoxia in the *A. fulgidus* growth medium, as previously described (Oliver et al., 2020; glass syringes were required at the higher incubation temperature for this strain to limit oxygen diffusion into the anoxic growth medium during incubation). To prevent syringe leakage at HT-HHP, custom-made butyl rubber stoppers served as an extra seal between the growth medium and the syringe piston. For incubation in both plastic and glass syringes with Luer lock fittings, the needle hub was embedded in butyl rubber stoppers before being transferred into the pressure vessels. Four High Pressure Equipment Co.© (HiP©), OC-1 O-Ring static pressure vessels were equipped with individual pressure gauges, and heating systems were similar to those described above for the PUSH vessels. Each 125 ml vessel held one 10 ml glass syringe. The temperature range of this system is 25°C–121°C and accommodates pressures from 0.1 to 100 MPa (based on the maximum working pressure capacities of the HiP© vessels and the temperature range of the BUNA O-Rings).

### Microbial Strains and Growth Medium

#### Selection of Target Strains for HT-HHP Growth

The choice of *D. salexigens* and *A. fulgidus* allowed for a comparison between two strains with similar metabolic strategies (heterotrophic sulfate reduction) over a range of temperatures (*D. salexigens* is a mesophile, while *A. fulgidus* is a thermophile) for two genera that are ubiquitous in subsurface environments and also represent both prokaryote. Each of these strains have had previous indications of growth at elevated pressure, belong to genera with other piezotolerant/piezophilic species, and also have been identified in high-pressure ecosystems. For example, a previous study, based on sulfide production measurements, showed that *D. salexigens* was active up to 5 MPa (Bale et al., 1997), and three other *Desulfovibrio* species have been isolated from subsurface environments (*D. profundus*, *D. hydrothermalis*, and *D. piezophiles*; Bale et al., 1997; Alazard et al., 2003; Khelaifia et al., 2011). While *A. fulgidus* type strain VC-16 was isolated from a shallow marine vent (Stetter et al., 1987, 1988), this strain has been identified in a number of deep-sea and deep subsurface environments (1–4 km and ~10–40 MPa; Stetter et al., 1987; Beeder et al., 1994; L'Haridon et al., 1995; Nakagawa et al., 2005; Fardeau et al., 2009). Additional species within the genus *Archaeoglobus* have been isolated from deep-sea environments (e.g., *Archaeoglobus veneficus* was isolated from the Mid Atlantic Ridge at 3.5 km depth, Huber et al., 1997), and archaeal sequences belonging to *Archaeoglobaceae* have been identified at the Mid-Cayman Rise, the deepest known hydrothermal system that reaches 4.96 km depths (~50 MPa; Reveillaud et al., 2016). Further, we have previously reported high-pressure growth of *A. fulgidus* (Oliver et al., 2020), showing that this strain is tolerant up to ~30–40 MPa (with decompression), which is consistent with the depths and pressures of deep-sea and deep subsurface environments where it has been identified.

#### Growth Conditions for *Desulfovibrio salexigens* and *Archaeoglobus fulgidus*

*Desulfovibrio salexigens* (DSM 2638), a marine sulfate-reducing bacterium (Postgate and Campbell, 1966) was obtained from the Deutsche Sammlung von Mikroorganismen und Zellkulturen (Germany) and grown anaerobically in DSMZ medium 163 (Solution A: NaCl 25 g L^−1^, K_2_HPO_4_ 0.5 g L^−1^, NH_4_Cl 1.0 g L^−1^, Na_2_SO_4_ 1.0 g L^−1^, CaCl_2_.2H_2_O 2.0 g L^−1^, MgSO_4_.7H_2_O 2.0 g L^−1^, Na-DL-lactate 2.0 g L^−1^, Yeast Extract 1.0 g L^−1^, resazurin solution (0.1% w/v) 0.5 ml L^−1^, distilled water 980 ml; Solution B: FeSO_4_.7H_2_O 0.5 g/10 ml and Solution C: Na-thioglycolate 0.1 g/10 ml, ascorbic acid 0.1 g/10 ml), similar to Postgate and Campbell (1966), with the exception that the media was enriched in calcium (1.5 times) and reduced in iron (1:100). Solutions B and C were added to solution A under N_2_ and the pH was adjusted to 7.8 with NaOH.

*Archaeoglobus fulgidus* strain VC-16 (DSM 4304) was obtained from the Deutsche Sammlung von Mikroorganismen und Zellkulturen GmbH (DSMZ, Braunschweig, Germany). *Archaeoglobus fulgidus* VC-16 is an anaerobic, hyperthermophilic sulfate-reducing archaeon. Here, *A. fulgidus* was grown chemolithoheterotrophically in a lactate–sulfate-rich medium. The composition of the culture medium followed Hartzell and Reed (2006) and sterile anaerobic conditions were maintained following Balch et al. (1979). The medium was reduced prior to inoculation by adding Na_2_S.9H_2_O to a final concentration of 0.1% prior to inoculation (Cario et al., 2016).

*Desulfovibrio salexigens* cultivations with or without decompression (static pressure vessels, and PUSH vessels, respectively) were tested at 0.1, 10, 20, 30, 40, and 50 MPa. Subsamples were taken at ~12 h intervals for 72 h to obtain a robust growth curve. For the higher pressures (30–50 MPa), a cell count after 150 h of growth was performed to enumerate cell density (death or survival) according to the pressure cultivation technique. Cell recovery experiments were performed for this strain in order to evaluate the impact of elevated pressure on cell growth recovery. The protocol and the obtained results are detailed in Table 1. *Archaeoglobus fulgidus* VC-16 was cultivated from 0.1 to 98 MPa in ~10 MPa increments at 83°C in PUSH vessels and in static pressure vessels. Six to eight subsamples were taken at regular time intervals for standard growth curves. *Archaeoglobus fulgidus* cells grown in the glass syringes were decompressed and repressurized a maximum of nine times throughout each batch culture experiment.

**Table 1:** The percentage of *D. salexigens* cell recovery after 36 h of growth at different elevated pressures, with or without decompression steps, and transfer at ambient pressure.

Cultivating pressures	Percentage of growth recovery after transfer at ambient pressure								
	Dilutions	After 24 h of growth				After 48 h of growth			
		Decompressed		No decompressed		Decompressed		No decompressed	
		%	SD	%	SD	%	SD	%	SD
10 MPa	1	50.7	1.7	90	2.2	76.7	12.5	99	0.5
	10	81.8	5.1	82	1.7	84.8	8.3	97	1.2
	100	52.5	17.2	81	3.6	95.5	3.9	99	0.8
20 MPa	1	20.2	2.2	76	2.8	69.1	14.8	98	1.1
	10	12.6	3.5	68	1.7	78.3	18.8	99	0.2
	100	10.2	3.7	71	5.2	64.2	15.1	99	0.8
30 MPa	1	NG		50	4.3	NG		89	2.5
	10			48	2.6			91	3.1
	100			55	3.8			85	5.8
40 MPa	1	NG		37	6.2	NG		80	5.2
	10			39	2.5			78	3.9
	100			31	4.8			81	8.7
50 MPa	1	NG		25	5.7	NG		78	10.2
	10			22	7.2			72	8.9
	100			17	12.3			70	15.2

The cell counts were measured after 24 and 48 h of growth at ambient pressure after the transfer from high-pressure to ambient pressure. For each pressure condition (P), one non-pressurized culture (ambient pressure) was used as a control (U). The average ratio of the growth recovery (U/P) was calculated for each dilution series and was performed at least in triplicate. SD, standard deviation and NG, no growth was observed. Significant differences were determined by Student’s *t*-test (value of *p* < 0.01).

### HT-HHP Culture Experiments

#### Pre-cultures and Inoculation

For triplicate HT-HHP culture experiments with and without sample decompression, the sterile media and pre-cultures were prepared for four PUSH vessels and four syringes incubated in static pressure vessels, corresponding to three inoculated culture replicates and one uninoculated experiment to serve as a negative control. Each strain was first grown from a frozen stock (−80°C) and transferred into sterile anaerobic growth medium. Exponential growth of this culture was used as inoculum for three independent pre-cultures. After reaching logarithmic-phase growth, each of these pre-cultures was used to inoculate fresh sterile growth medium in three serum bottles. Immediately following inoculation, 8–10 ml from each inoculated serum bottle were transferred into sterile syringes and the remaining 45–47 ml were transferred into each PUSH vessel. The *D. salexigens* culture started at 1.10^6^ cells/ml and incubations were carried out in static pressure vessels and PUSH vessels at 30°C. Inoculation and transfer of *A. fulgidus* cultures were carried out in the anaerobic chamber in pre-heated PUSH vessels. Starting cell concentrations for *A. fulgidus* experiments were ~6.7 × 10^6^ cells/ml, and incubations were carried out at 83°C.

#### PUSH Vessel Preparation, Pressurization, and Subsampling

For each batch culture HT-HHP experiment, four PUSH vessels were sterilized, assembled, and pre-heated before inoculation and transfer (Supplementary Information 1, Supplementary Figure S1). After pre-assembly, each PUSH was individually wrapped in an insulated temperature-controlled system with thermocouple and heating jacket to 30°C and 83°C, for *D. salexigens* and *A. fulgidus*, respectively (Supplementary Information 1, Supplementary Figure S1B). Once pre-heated, all four vessels were transferred with their respective temperature-control systems into an anaerobic chamber (Bactron Shellab), and each temperature-control system was plugged into the DC to AC power converter allowing for continual vessel heating throughout the anaerobic inoculation process. In the anaerobic chamber, the PEEK reservoirs of each of the four PUSH vessels were filled with ~45–47 ml of pre-inoculated growth medium (triplicate) or sterile growth medium (Supplementary Information 1, Supplementary Figure S1C). The PEEK reservoir screw cap and lid was then closed completely and the valves were closed while in the anaerobic chamber. The vessels were then transported out of the anaerobic chamber for pressurization.

Each PUSH vessel was individually pressurized to the target pressure with a hydraulic screw pump with an in-line pressure gauge connected to the PEEK piston valve (Supplementary Information 1, Supplementary Figure S1D). Once pressurized, an initial 0.5 ml subsample was taken and fixed in 2.5% glutaraldehyde from each PUSH vessel using the decompression line (see below). Initial sampling and pressurization of the all of the PUSH vessels and syringes were done within 1 h of inoculation in the anaerobic chamber. To assure pressure stability, potential pressure loss from vessel leakage was closely monitored for the first 3 h after inoculation. Up to nine subsamples were obtained from each PUSH vessel for every HT-HHP batch culture growth experiment. For subsampling, the average decompression rate was of 15–25 MPa/min (Supplementary Information 1, Supplementary Figure S1E). The first 3 ml of medium sampled were discarded as waste from flushing the decompression line before taking a 0.5 ml aliquot sample for enumeration. A maximum of 10% pressure loss occurred during subsampling, but in all cases the pressure was rapidly re-established. The decompression line was cleaned with 70% ethanol and ultrapure water (18.2 MΩ) before and after subsampling each PUSH vessel.

#### Syringe Preparation, Pressurization, and Subsampling

For both strains, *D. salexigens* and *A. fulgidus*, four 10 ml syringes were used for HT-HHP batch cultivation with sample decompression. Ten milliliter sterile plastic syringes were used for *D. salexigens* and 10 ml glass syringes were used for *A. fulgidus*. Both plastic and glass syringes were flushed with N_2_ and the syringe needles were then embedded into butyl stoppers (Supplementary Figure S2B). The assembled syringes were then inoculated in the anaerobic chamber by transferring 8–10 ml of inoculated medium into each of the three 10 ml glass/or plastic syringes. For a negative control, 8–10 ml of sterile medium was transferred in the fourth 10 ml glass/or plastic syringe (Supplementary Information 2 and Supplementary Figure S2C). The syringes were removed from the anaerobic chamber and an initial 0.5 ml subsample was fixed in 2.5% glutaraldehyde for enumeration. Finally, each syringe was placed in one of the four pre-heated HiP© vessels, filled with water, and pressurized by connecting each vessel to a HHP screw pump to obtain the target growth pressure (Supplementary Information 2, Supplementary Figure S2D). For syringe subsampling, each vessel was decompressed at an average rate of 19 MPa/min and a 0.5 ml aliquot sample was taken and fixed in 2.5% glutaraldehyde from each syringe. After subsampling, the syringes were returned to the vessels and again pressurized to the target growth pressure.

### Light Microscopy and Cell Enumeration

Cell enumeration was estimated by direct counts from fixed cells [2.5% (v/v) glutaraldehyde] in a Thoma-chamber (depth: 0.02 mm; Brand, Wertheim, Germany) using a light microscope (model XM: Olympus) under 80x magnification (e.g., Huber et al., 1989; Hei and Clark, 1994; Blöchl et al., 1997; Cario et al., 2016). Specific growth rates (μ^-hr^) were calculated from linear regressions of the exponential portion of the growth curves from triplicate experiments using the LINEST function in Excel. Error bars indicate the standard error from linear regressions of triplicate experiments. Cell densities quantified at ~36 h for *A. fulgidus* and 48 h for *D. salexigens* were used to evaluate the effects of different cultivation conditions on overall cell yields. As some pressure conditions led to cell death, maximum cell densities could not be used for comparison. Error bars indicate SD from triplicate experiments. Significant differences were determined by Student’s *t*-test (value of *p* < 0.01).

## Results

### *Desulfovibrio salexigens* Growth at Elevated Pressure

**Figure 1:**
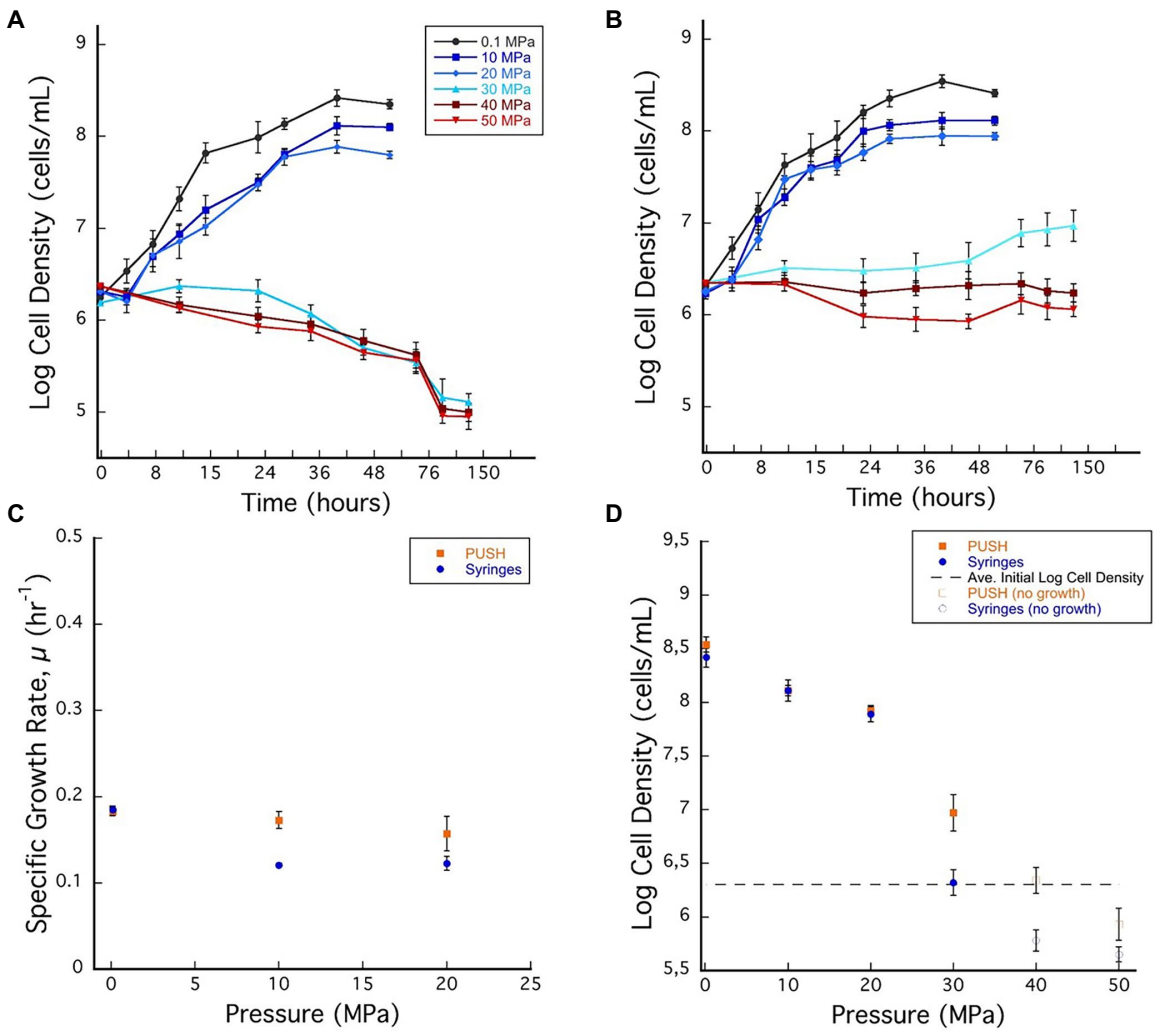
**(A)** *Desulfovibrio salexigens* growth curves in plastic syringes with multiple sample decompression and **(B)** *D. salexigens* growth curves in the pressurized underwater sample handler (PUSH) vessels without multiple sample decompression from 0.1 to 50 MPa. **(C)** *Desulfovibrio salexigens* specific growth rates in the PUSH vessels (orange squares) and in syringes (blue circles). **(D)** *Desulfovibrio salexigens* maximum log cell densities in the PUSH vessels (orange squares) and in syringes (blue circles), open orange squares and blue circle indicate maximum cell densities in samples with no observed growth or observed cell densities lower than the average initial cell densities (dashed lines in **D**). Error bars are the SDs from the average of triplicate experiments. Significant differences were determined by Student’s *t*-test (value of *p* < 0.01).

Elevated pressure experiments were performed from ambient (0.1 MPa) up to 50 MPa for *D. salexigens* using both static pressure vessels (i.e., with cyclic decompression) and variable volume PUSH vessels (i.e., isobaric) for experiments that lasted 76–150 h. Growth curves, growth rates, and cell density data are summarized in Figure 1, Supplementary Table S1. At some elevated pressures, cell numbers decreased with time (e.g., cell death). To account for both cell growth and cell death at different conditions, cell densities are compared at 48 h over the entire pressure range of the experiments (Figure 1D).

Exponential growth of *D. salexigens* was observed up to 20 MPa both in the static and PUSH vessels (Figures 1A,B). The highest growth rates were observed at ambient pressure (0.1 MPa) and 30°C, and were nearly identical for cultivation in serum bottles and the PUSH vessels (0.19 ± 0.005 h^−1^ and 0.19 ± 0.004 h^−1^, respectively). The same growth rate was confirmed with *D. salexigens* incubation, performed in syringes, at ambient pressure and 30°C (data not shown). Overall growth rates decreased with pressure, but declining growth rates were less pronounced for isobaric enrichments (Figure 1C; Supplementary Table S1). For example, at 10 MPa the growth rate for isobaric cultivation was 0.17 ± 0.2 h^−1^, ~10.5% lower than optimal conditions, while cultivation with decompression had a ~36.8% drop in growth rate to 0.12 ± 0.1 h^−1^. Furthermore, the upper pressure limit for growth was higher in isobaric experiments, with slow growth observed at 30 MPa in PUSH vessels (0.02 h^−1^), compared to distinct cell death in decompressed experiments (Figures 1A–C).

While differences in growth rates were notable between the two cultivation techniques at moderate pressures (0.1–20 MPa), there were negligible effects on cell yields in this pressure range (Figure 1D; Supplementary Table S1). However, at supra-optimal pressures (≥30 MPa for *D. salexigens*), differences in cell densities (quantified at 48 h) were more significant. For cultivation experiments that included cyclic decompression, cell numbers decreased markedly with time at 30, 40, and 50 MPa, indicating cell death. Conversely, isobaric cultivation at these pressures led to only minor changes in cell density with time. For example, at 40 MPa, cell density decreased from an initial value of 10^6.30^–10^5.78^ after 48 h, while the isobaric culture had a cell density of 10^6.34^ after 48 h and no decompression (Figure 1D). When considering changes in cell yields, decompression had a more significant impact at supra-optimal pressures, inducing cell death, as opposed to isobaric conditions, which only limited growth.

To further explore the impact of decompression on *D. salexigens* cell viability, cells were incubated at various elevated pressures (10–50 MPa) for 36 h, with or without decompression. They were then transferred to ambient pressure, incubated in fresh growth medium at several dilutions (1:1, 1:10, and 1:100, v/v), and cell recovery was estimated after 24 and 48 h of growth. Because the syringe cultivation technique requires decompression of the high-pressure vessels for subsampling, each vessel was subjected to six decompression/repressurization cycles with a minimum of 4 h between cycles. For both static pressure vessels and PUSH vessels, cells from the 10 and 20 MPa growth conditions were able to recover the optimal pressure cell density (see Table 1). However, when the cells were incubated at higher pressure (≥30 MPa) with cyclic decompression the cells were not able to recover and did not grow after 24 or 48 h of transfer at ambient pressure (nor after 1 week of incubation, data not shown). In comparison, cells exposed to 30–50 MPa in the PUSH without decompression remained viable, though were slower to recover as pressures increased. After transfer to and growth at ambient pressure (0.1 MPa) for 48 h, cell densities were only slightly lower (90% for 30 MPa and 70% for 50 MPa) than those observed for cells never exposed to elevated pressure (Table 1).

### *Archaeoglobus fulgidus* Growth at Elevated Pressure

**Figure 2:**
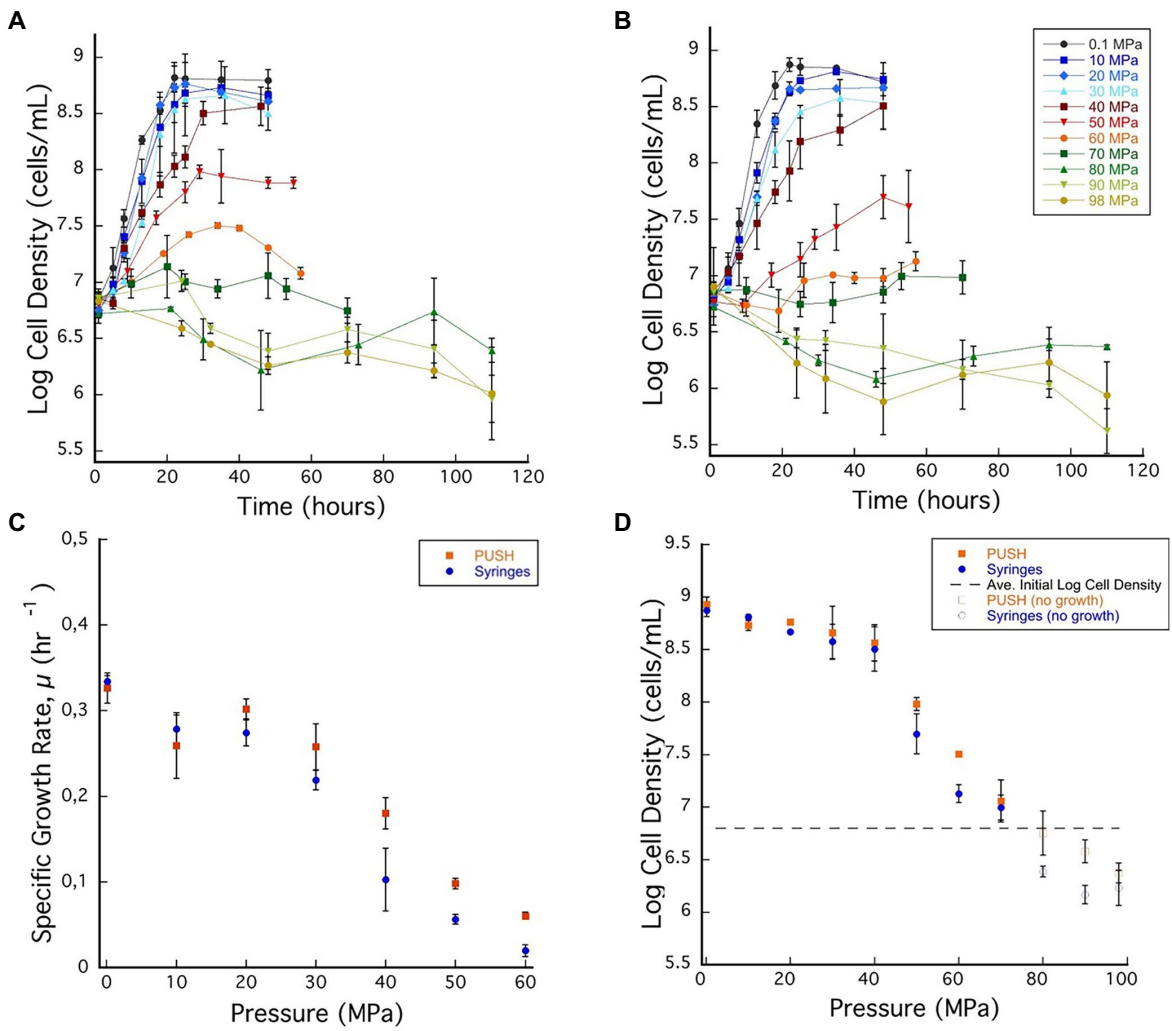
**(A)** *Archaeoglobus fulgidus* growth curves in glass syringes with multiple sample decompression and **(B)** *A. fulgidus* growth curves in the PUSH vessels without multiple sample decompression from 0.1 to 98 MPa. **(C)** *Archaeoglobus fulgidus* specific growth rates in the PUSH vessels (orange squares) and in syringes (blue circles). **(D)** *Archaeoglobus fulgidus* maximum log cell densities in the PUSH vessels (orange squares) and in syringes (blue circles), open orange squares and blue circle indicate maximum cell densities in samples with no observed growth or observed cell densities lower than the average initial cell densities (dashed lines in **D**). Error bars are the SDs from the average of triplicate experiments. Significant differences were determined by Student’s *t*-test (value of *p* < 0.01).

*Archaeoglobus fulgidus* VC-16 (type strain) was grown in batch cultivation experiments at 83°C over a range of pressures (0.1–98 MPa, at ~10 MPa increments) in PUSH vessels (isobaric) and in static pressure vessels (cyclic decompression) and cell densities were monitored up to 115 h. Growth curves, growth rates, and cell density data are summarized in Figure 2; Supplementary Table S1. To assess the impact of decompression on cell yields even in the cases of cell death, cell densities at 36 h are reported over the entire pressure range (Figure 2D). This time aligns with the generally faster growth of this strain, captures the exponential phase at lower pressures, and accurately represents cell death at elevated pressures.

Based on the growth curves shown in Figures 2A,B, *A. fulgidus* growth rates were calculated for pressures between 0.1 and 60 MPa (Figure 2C). Overall, *A. fulgidus* growth characteristics showed trends with pressure similar to those observed for *D. salexigens.* Exponential growth was observed in the PUSH vessels at 60 MPa until ~29 h, after which cell numbers decreased. In static pressure vessels at this pressure, only minor increases in cell density (1.34 ± 0.11 × 10^7^ cells/ml) relative to initial values (6.03 × 10^6^ ± 1.02 cells/ml) were observed, but were not consistent with a true exponential growth phase (Figure 2A). Growth was generally faster with higher maximum cell densities (with the exception at 10 MPa) in PUSH vessels compared to static pressure vessels, especially at the higher end of the pressure range (Figures 2C,D). The fastest growth rate in the PUSH vessels was measured at 0.1 MPa (0.326 ± 0.017 μ^-hr^), though growth rates were only slightly slower from 10 to 30 MPa (0.258 ± 0.026–0.302 ± 0.011 μ^-hr^). The largest difference in growth rates between the two cultivation methods was observed at 30 and 40 MPa (15.3% and 44%, respectively). Overall, growth rates indicate that *A. fulgidus* VC-16 is piezosensitive with a maximum exponential growth pressure of 60 MPa in isobaric conditions and 50 MPa for cultivation that included cyclic decompression.

Similar to the mesophilic strain, *D. salexigens*, the two cultivation techniques had a more significant impact on *A. fulgidus* cell density in the supra-optimal pressure range (≥50 MPa), while density was largely unaffected at the lower pressures. For example, from 0.1 to 40 MPa the largest difference in density at ~36 h was at 30 MPa (10^8.66^, isobaric vs. 10^8.56^, decompressed). Comparing isobaric growth to cultivation with cyclic decompression shows that the largest disparities in cell density (measured at ~36 h) were observed at 60 MPa when growth was observed and at 90 MPa when only cell death was recorded. Again, these data suggest that cycles of decompressions can accelerate loss of cell viability. Loss of cell viability was also tested *via* high-pressure incubation experiments in which *A. fulgidus* cells were incubated at 80 MPa for 115 h and subsequently transferred (10% v/v) to fresh growth medium and incubated at 0.1 MPa and 83°C. Ambient pressure cultures were monitored visually and no growth was observed after a week of incubation.

### Pressure-Induced Effects on Cell Mobility and Morphology

We also observed significant effects of elevated pressure on the mobility and the morphology of *D. salexigens* cells using a light microscope (Supplementary Figure S3). Under optimal pressure conditions (i.e., ambient pressure, 0.1 MPa), the cells were highly motile and formed 3–4 μm vibrios and up to 20 μm cell long when reaching the late exponential and stationary phases (Supplementary Figure S3). Under high-pressure cultivation conditions (10 and 20 MPa) and subsequent sampling, the cells were barely motile and formed short vibrios (2–3 μm). After longer incubation times they also formed filamentous cells (Supplementary Figure S1). At higher cultivation pressures without decompression steps (30–50 MPa) and subsequent sampling, the cells were also immotile. When they were transferred for growth at ambient pressure, the cell division appeared altered with formation of cells arranged in chains (Supplementary Figure S4). Irregular and elongated cell morphologies were previously observed in *A. fulgidus* at elevated pressures (Oliver et al., 2020). This morphological feature has been reported in several studies, where elevated pressure inhibited the cell division protein FtsZ (e.g., Ishii et al., 2004; Abe et al., 2013). Morphological changes are a common stress response (e.g., Zobell and Oppenheimer, 1950; Donaldson et al., 1989) and might be a global stress response to high-pressure (e.g., Bidle and Bartlett, 1999; Aertsen et al., 2004).

Additionally, for elevated pressure conditions (10–50 MPa) we noticed the presence of cyst-like cells (Supplementary Figure S3), a constitutive dormancy behavior characteristic of some non-spore forming bacteria (Suzina et al., 2004). As pressure increased from 10 to 50 MPa, we observed an increasing abundance (approximately 2-fold) of such pleiomorphic cells (Supplementary Figure S5). However, after transferring these cultures to ambient pressure, we observed the release of cells resembling the development of vegetative cells, suggesting that these structures could be cysts (Supplementary Figure S4). The formation of various types of structures, such as endospores, exospores, or cysts, is previously recognized survival strategy of many bacteria under unfavorable conditions induce (Sudo and Dworkin, 1973). The pressures at which we observed changes morphologies and structures are consistent with the growth data that indicated the onset global stress, as indicated by slower growth rates and decreasing cell numbers. Interestingly, among the dissimilatory sulfate-reducing bacteria, the genus *Desulfovibrio* was characterized as non-sporulating compared to the closely related genus *Desulfotomaculum* (Postgate and Campbell, 1966). Such behavior might help this species to cope with extreme growth conditions, such as elevated pressure conditions, and might be an important contribution for microbial dissemination in various environments. These specific structures deserve more investigations to determine the developmental steps and the ultrastructural properties of this species when growing under unfavorable growth conditions.

## Discussion

A majority of the Earth’s biosphere thrives in high-pressure, subsurface environments (Fang et al., 2010; Oger and Jebbar, 2010; Picard and Daniel, 2013), and like many natural ecosystems, studies of subsurface diversity reveal a vast number of uncultured species, often only identified through the presence of their genetic material. The difference between the diversity observed in molecular studies and the strains cultivated from natural systems is likely due to the loss of cell viability during sampling, or the enrichment and isolation protocols. The importance of using growth media compositions to target unique metabolic strategies and thus increase the diversity of cultured species has been discussed elsewhere (e.g., Alain and Querellou, 2009; Overmann et al., 2017). Furthermore, the idea of reducing sample exposure to conditions that cause cell death is regularly used, as is the case for obligate anaerobes, specifically methanogens. More recently, similar reasoning has been applied to the high-pressure biosphere; with novel technologies being deployed to capture and characterize microbes at elevated pressure (e.g., Garel et al., 2019; Peoples et al., 2019). Nonetheless, widespread application of high-pressure techniques throughout sampling, transfer, enrichment, and isolation is limited by challenges associated with cost, technology development, and technical expertise that are amplified when considering high-pressure sampling and cultivation (Cario et al., 2019). Furthermore, even when enrichments or isolates are cultivated at elevated pressures in laboratory settings, traditional methods that use static pressure vessels require decompression during subsampling (e.g., Takai et al., 2008), and the effects of decompression on experimental results for laboratory cultivation experiments has not been thoroughly or systematically investigated.

To explore the potential effects of decompression on microbial growth dynamics, we used two model sulfate reducers, *D. salexigens* and *A. fulgidus* to compare growth rates, cell yields, and maximum growth pressures between cultivation experiments that included several cycles of decompression and repressurization during subsampling, and those that maintained cultures at constant pressure throughout. Both strains showed significant exponential growth at elevated pressure for both types of cultivation conditions, although both had maximum growth rates at ambient pressure, classifying them as piezosensitive strains. We note that in previous studies we observed piezophilic behavior with maximum growth rates at 20 MPa (Oliver et al., 2020), and found that the growth rate measured at 0.1 MPa was particularly sensitive to the presence of a gas phase. Additionally, the maximum pressure for exponential growth for both strains was extended under isobaric conditions, confirming that cultivation techniques can impact experimentally determined growth characteristics of isolated strains.

In general, the impacts of the two cultivation techniques (isobaric vs. cyclic decompression) followed similar patterns for both *D. salexigens* and *A. fulgidus*, even though the specific pressure ranges, growth rates, and cell yields were strain-specific. Comparing cultivation techniques across both species, growth dynamics can be delineated into three different pressure regimes. The low-pressure regime (LP) has robust exponential growth with only marginal decreases in growth rates as pressures increase and little variation in maximum cell density. The high-pressure regime (HP) includes the range that exceeds the maximum pressure for exponential growth and is often characterized by cell death. The transitional pressure regime (TP) lies in between, is characterized by markedly lower growth rates and cell yields than those observed in the LP, and sometimes exponential growth is hard to discern. Within the TP the impacts of several cycles of decompression and repressurization are most noticeable for both growth rates and maximum cell yields.

The LP regime of *D. salexigens* is 0.1–20 and 0.1–40 MPa for *A. fulgidus.* For both strains, exponential growth rates decreased with increasing pressure, but maximum cell densities were similar at the end of the exponential growth phase. In this low-pressure regime, the effects of cyclic decompression are pronounced for growth rates, which are lower for cyclic decompression cultivation compares to isobaric experiments. However, little to no discernible impact was observed for cell densities. Growth rates and cell densities are more variable in the TP and HP regimes. For *D. salexigens* the transition pressure range is 30–40 MPa. While exponential growth is not discernible at 30 MPa for either condition, there are measurable increases in cell density late in the isobaric cultivation, while only cell death is observed with cyclic decompression cultivation (Figure 1A). It is also in the TP range that the largest difference in cell density is observed (Figure 1D). A similar pattern is observed for *A. fulgidus* for which the transition pressure range is 40–70 MPa. Exponential growth is observed in isobaric cultures up to 60 MPa, while the limit for cell division in cyclic decompression cultivation is 50 MPa. Overall, significantly slower growth rates (Figure 2C) were observed in *A. fulgidus* subjected to decompression from 40 to 60 MPa. Furthermore, the impact of decompression on cell density was also most apparent at 50 and 60 MPa (Figure 2D). Finally, while the HP regime is generally characterized by cell death (decreasing cell density with time) there are still noticeable differences between the two cultivation techniques. For *D. salexigens* no growth was obvious at 40 or 50 MPa, however cells subjected to cyclic decompression exhibited clearly higher rates of cell death (Figure 1A). Additionally for *A. fulgidus*, decreases in cell density with time were slower for isobaric cultivation.

The processes that might cause the largest differences in both growth rate and cell yield in the TP and HP are not immediately apparent. However, the robust exponential growth in the LP indicates that cells in this lower pressure range are not significantly impacted by these conditions. As both *D. salexigens* and *A. fulgidus* are piezosensitive strains that were isolated and optimized at ambient pressure conditions (0.1 MPa), it follows that decompression from moderate pressures to ambient pressure (their optimum pressure for growth) has little impact on growth rates and cell yields. However, for piezotolerant and piezophilic organisms, decompression would repeatedly expose cells to sub-optimal pressure conditions. In these cases, we hypothesize that cyclic decompression would have a more significant negative impact on growth. Additionally, if extended exposure to elevated pressure were inducing adaptation, even in a subpopulation of the culture, these cells would be selected against during decompression. Further characterization of these and other piezotolerant and piezophilic strains are critical next steps to better understand the effects of cyclic decompression on laboratory characterization of isolated species. Overall, we expect that the difference between isobaric cultivation and cultivation with cyclic decompression will be more pronounced in piezotolerant and piezophilic strains.

## Concluding Remarks

For two deep biosphere species investigated here, *A. fulgidus* and *D. salexigens*, growth was negatively impacted by sample decompression, and these detriments to growth were more significant at pressures further from optimum pressures. These results highlight the need to maintain constant pressure without decompression during cultivation of high-pressure strains. Such an approach is likely to increase the pressure ranges of piezotolerant species, potentially reclassify other strains as piezophilic, and ultimately expand our knowledge of the diversity of known piezophiles, putting into perspective the habitat constraints for many strains whose pressure ranges have been underestimated. Even the piezophilic and piezotolerant organisms that are in culture have likely been isolated after decompression, inevitably selecting against strains more sensitive to decompression. For high-pressure samples that are decompressed and subsequently enriched at elevated *in situ* pressures, those strains that are somewhat tolerant to decompression, like *A. fulgidus*, would outcompete more sensitive strains, and results of any subsequent analyses would not necessarily be representative of the active *in situ* microbial communities.

Furthermore, maintaining *in situ* pressures becomes even more critical when retrieving samples from deep ecosystems, for example, 5–6 km—habitats more likely to host obligate piezophiles—for which decompression would subject native communities to pressure changes of over 50 MPa. Thus far, most of the obligate piezophiles identified have been psychrophilic bacteria sampled from depths greater that 6 km (i.e., ~60 MPa pressures; for example, Yayanos et al., 1981; Deming et al., 1988; Kato et al., 1998; Nogi et al., 2004) and only one obligate piezophilic hyperthermophilic archaeon, *Pyrococcus yayanosii*, has been identified from a hydrothermal vent at 4.1 km depth (Birrien et al., 2011). Given the extent of the subsurface biosphere, it is unlikely that these few obligate piezophiles represent the full diversity of Earth’s largest microbiome. Variable volume, high-pressure devices like the PUSH and others (reviewed in Cario et al., 2019), can be used not only to retrieve samples from up to ~9–10 km water depth, but all can be connected in series (as shown here and in Garel et al., 2019), so that sample transfer and enrichment can also be carried out without decompression. Such an approach will be more selective for piezophiles and obligate piezophiles, and greatly expand our understanding of the activity and physiology of the deep biosphere microbiome.

## Data Availability Statement

The original contributions presented in the study are included in the article/Supplementary Material; further inquiries can be directed to the corresponding authors.

## Author Contributions

GO, AC, and KR designed this research project and analyzed data. GO and AC performed the experiments and collected data and wrote the original draft. KR edited and wrote sections of the manuscript. All authors contributed to revisions of the manuscript, tables, and figures and approved the submitted version.

## Funding

Funding for this work was provided by the NASA Exobiology and PSTAR Programs (NNX13AP2G9 and 80NSSC17K0252 to KR), the Deep Carbon Observatory (Subawards: 10371-07, 10561-01, and 10311-11 to KR), an NSF Graduate Fellowship (FAIN 1247271 and 1744655 to GO), and a GSA Research grant to GO. Additional support was provided by startup funds from Rensselaer Polytechnic Institute to KR.

## Conflict of Interest

The authors declare that the research was conducted in the absence of any commercial or financial relationships that could be construed as a potential conflict of interest.

## Publisher’s Note

All claims expressed in this article are solely those of the authors and do not necessarily represent those of their affiliated organizations, or those of the publisher, the editors and the reviewers. Any product that may be evaluated in this article, or claim that may be made by its manufacturer, is not guaranteed or endorsed by the publisher.
