# Supplementary material for: Characterizing the Piezosphere: The Effects of Decompression on Microbial Growth Dynamics
Source: Front Microbiol. 2022 May 17;13:867340. doi: 10.3389/fmicb.2022.867340 (PMC9157427; doi:10.3389/fmicb.2022.867340)
Supplement: Supplementary file 1 [file Data_Sheet_1.pdf]

Supplementary information 1: PUSH vessel preparation, pressurization and subsampling

To sterilize the PUSH vessels, each vessel was partially assembled with the PEEK reservoir inserted into the PUSH vessel body and the PEEK piston screw cap was attached (Fig. S1 A). The PEEK reservoir screw cap and lid was separately wrapped in foil. The valve connections and open PEEK reservoirs were also covered in foil. The foil-wrapped vessels, screw caps, and connections were sterilized by autoclaving at 121°C for 15 minutes. After sterilization, PUSH vessels were pre-assembled in a biosafety cabinet to maintain sterile conditions. The PEEK reservoir screw cap was left partially unthreaded so that it could be removed later to fill the reservoir with inoculated growth medium in the anaerobic chamber. Finally, valves were attached to each side of the PUSH vessel.

After pre-assembly, each PUSH was individually wrapped in an insulated temperature-controlled system with thermocouple and heating jacket to 30°C and 83°C, for *D. salexigens* and *A. fulgidus*, respectively (Fig. S1 B). Once pre-heated, all four vessels were transferred with their respective temperature-control systems into an anaerobic chamber (Bactron Shellab). In the anaerobic chamber, the four PUSH temperature-control systems were plugged into the DC to AC power converter allowing for continual vessel heating throughout the anaerobic inoculation process.

In the anaerobic chamber, the PEEK reservoirs of each of the four PUSH vessels were filled with ~45-47 mL of pre-inoculated growth medium (triplicate) or sterile growth medium (Fig. S1 C). The PEEK reservoir screw cap and lid was then closed completely and the valves were closed while in the anaerobic chamber. The vessels were then transported out of the anaerobic chamber for pressurization. For *A. fulgidus* high-temperature experiments, the vessels lost a maximum of 15°C during transfer in and out of the anaerobic chamber but returned to the target temperature (83°C) within 3-5 minutes after reconnection to the thermal control. Each vessel was then pressurized to the target pressure. For *A. fulgidus* high-temperature experiments, the vessels lost a maximum of 15°C during transfer in and out of the anaerobic chamber but returned to the target temperature (83°C) within 3-5 minutes after reconnection to the thermal control.

For subsampling, the HHP screw pump was connected to the PEEK piston valve and pressurized to the pressure of the vessel. Once pressurized, the PEEK piston valve was opened. Next, the decompression line with a sterile syringe attached was connected to the PEEK reservoir valve. The valves on the decompression line were closed. The PEEK reservoir valve was first opened and any pressure loss was regained using the HHP screw pump. The first valve on the decompression line was opened and again the target pressure was re-established. Finally, the microvalve was opened with an average decompression rate of 15-25 MPa/minute (Fig. S1 E). The first 3 mL of medium sampled were discarded as waste from flushing the decompression line before taking a 0.5 mL aliquot sample for enumeration. A maximum of 10% pressure loss occurred during subsampling in all experiments but in all cases the pressure was rapidly re-established. The decompression line was cleaned with 70% ethanol and ultrapure water (18.2 MΩ) before and after subsampling each PUSH vessel.

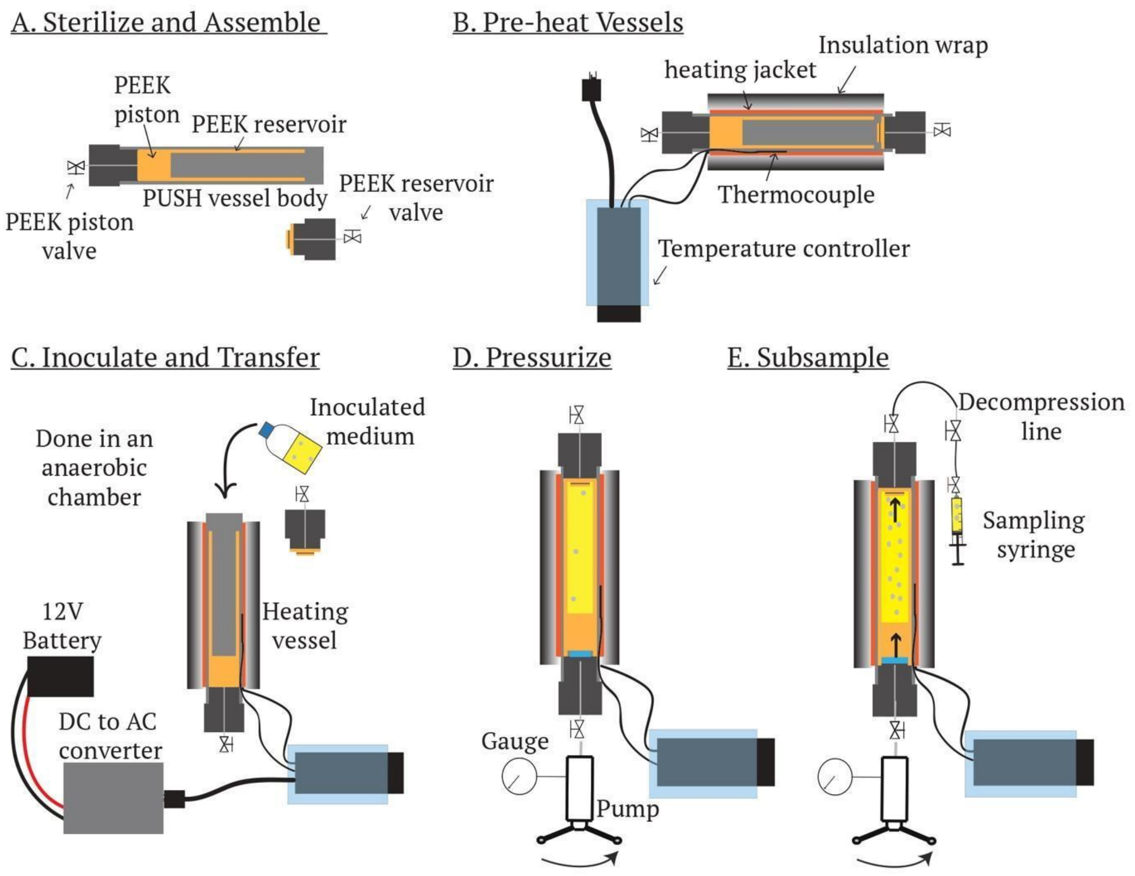

Figure S1. A schematic showing the procedures performed for preparing and running an *A. fulgidus* HT-HHP batch cultivation experiment in the PUSH vessels and temperature-controlled systems.

Supplementary information 2: Syringes preparation

To prepare the syringes for the experiment, the glass syringes, the syringe pistons, custom-made butyl stoppers, and needles were sterilized under a UV lamp for one hour in a biosafety cabinet (Fig. S2 A). After sterilization, the syringes were assembled in the biosafety cabinet, then, flushed with N_2 and embedded into butyl stoppers (Fig. S2 B).

The assembled syringes were then transported to the anaerobic chamber. Once in the anaerobic chamber and after inoculation, 8-10 mL of inoculated medium was transferred into each of the three 10 mL glass/or plastic syringes and 8-10 mL of sterile medium was transferred in the fourth 10 mL glass/or plastic syringe as a negative control (Fig. S2 C). The syringes were removed from the anaerobic chamber and an initial 0.5 mL subsample was fixed in 2.5% gludaraldehyde for enumeration. Finally, each syringe was placed in one of the four available preheated HiP© vessels, filled with water, and pressurized by connecting each vessel to a HHP screw pump to obtain the target growth pressure (Fig. S2 D).

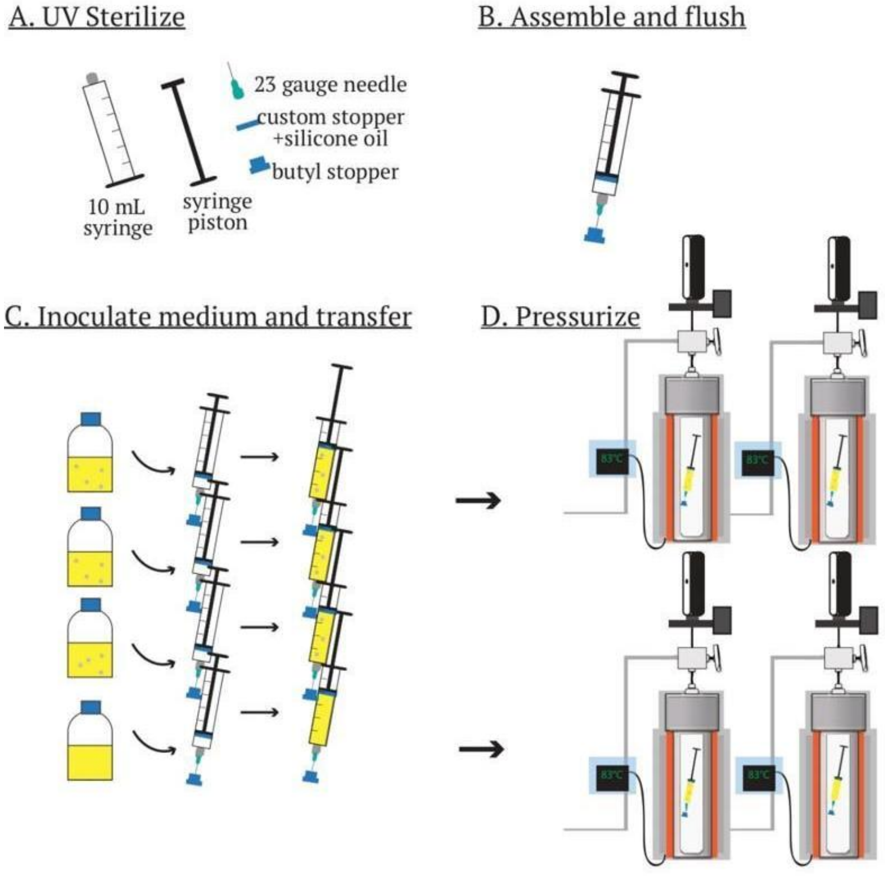

Figure S2. Glass syringe sterilization (A) and assembly (B) for batch culture growth experiments with sample decompression. Following inoculation, *A. fulgidus* cultures were transferred into 5 mL syringes (C) and placed in heated static pressure vessels filled with water and pressurized (D).

<u>Supplementary information 3: Figure S3</u>

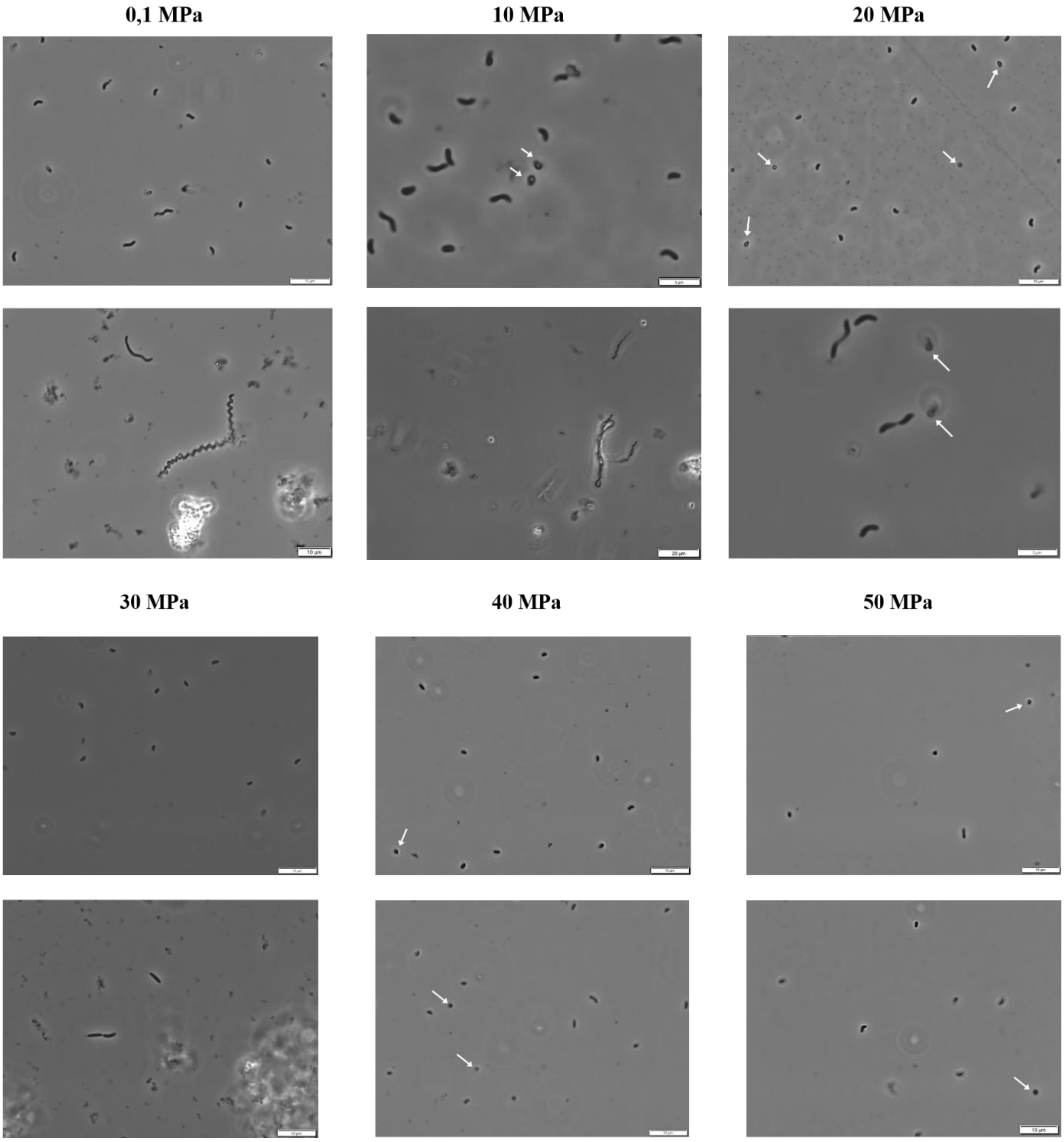

<u>Figure S3:</u> Pictures of *D. salexigens* with phase-contrast microscopy at different cultivating pressures. The white arrows indicate the presence of cyst-like cells. At 0.1 MPa and 10 MPa, the top pictures are cells in exponential phase of growth and the bottom ones, cells in stationary phase of growth. Elevated pressure conditions were performed without decompression steps.

<u>Supplementary information 4: Figure S4</u>

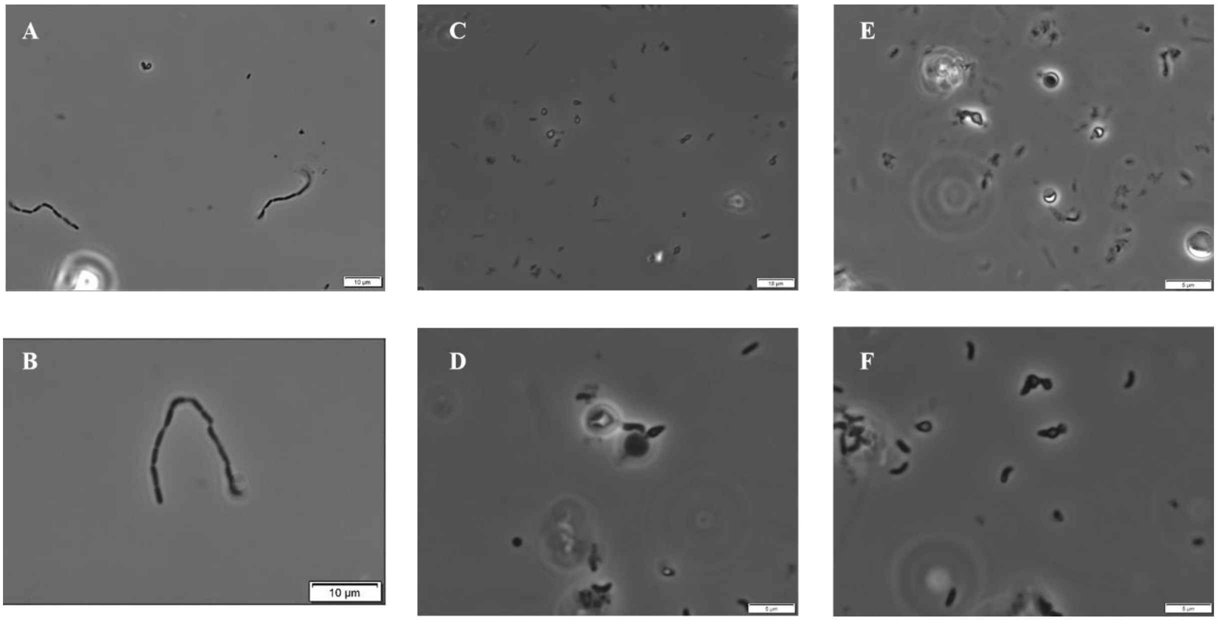

<u>Figure S4:</u> Pictures of *D. salexigens* after being transferred for growth at HP to ambient pressure. A and B show cells arranged in chains. C to F show the development of vegetative-like cells.

<u>Supplementary information 5: Figure S5</u>

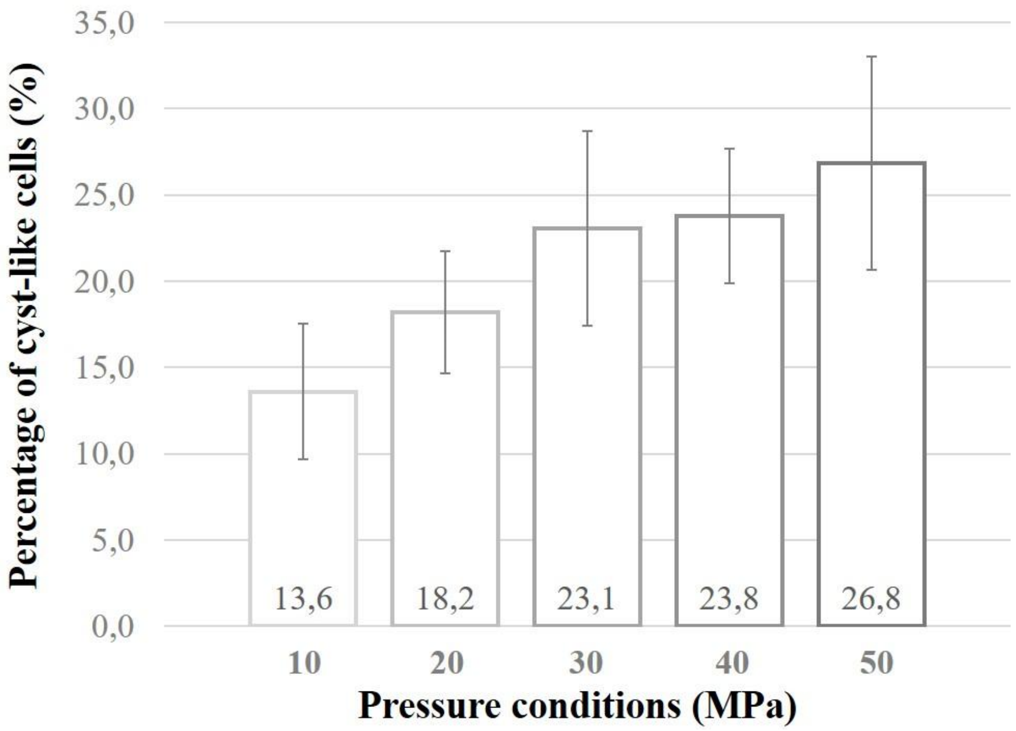

<u>Figure S5:</u> Percentage of cyst-like cells in the PUSH (without decompression) after growth at different pressure conditions (10 to 50 MPa). The error bars represent standard deviation from at least 10 counts on 10 different experiments. Significant differences were determined by Student's t test (p-value<0.01).

Supplementary information 6: Table S1

Strains	Pressure conditions (MPa)	Growth rate		Max log_{10} cell density	
		Isobaric	Decompressed	Isobaric	Decompressed
D. salexigens	0.1	0.18 ± 0.005	0.19 ± 0.004	8.54 ± 0.07	8.42 ± 0.09
	10	0.17 ± 0.01	0.12 ± 0.002	8.11 ± 0.05	8.11 ± 0.10
	20	0.15 ± 0.02	0.12 ± 0.008	7.92 ± 0.05	7.89 ± 0.07
	30	-	-	6.97 ± 0.17	6.32 ± 0.12
	40	-	-	6.34 ± 0.12	5.78 ± 0.10
	50	-	-	5.93 ± 0.15	5.65 ± 0.07
A.fulgidus	0.1	0.33 ± 0.02	0.33 ± 0.007	8.93 ± 0.07	8.87 ± 0.06
	10	0.26 ± 0.04	0.28 ± 0.02	8.73 ± 0.05	8.81 ± 0.02
	20	0.30 ± 0.01	0.27 ± 0.01	8.69 ± 0.02	8.65 ± 0.0003
	30	0.26 ± 0.03	0.22 ± 0.01	8.66 ± 0.25	8.56 ± 0.16
	40	0.18 ± 0.02	0.10 ± 0.04	8.50 ± 0.10	8.29 ± 0.13
	50	0.10 ± 0.006	0.06 ± 0.005	7.94 ± 0.24	7.43 ± 0.20
	60	0.06 ± 0.004	0.02 ± 0.007	7.50 ± 0.03	7.01 ± 0.01
	70	-	-	6.94 ± 0.08	6.76 ± 0.18
	80	-	-	6.59 ± 0.18	6.42 ± 0.05
	90	-	-	6.49 ± 0.04	6.25 ± 0.09
	98	-	-	6.44 ± 0.01	6.08 ± 0.06

Table S1: Summary of the growth data for both strains (Fig. 1 and 2) according to the pressure conditions and the high-pressure cultivation techniques, cyclic decompression or isobaric in the PUSH vessels. Significant differences were determined by Student's t test (p-value < 0.01).
